# An Edge-Computing Sensor Platform for ISO 2631-1 Whole-Body Vibration Exposure Metrics

**DOI:** 10.3390/s26185937

**Published:** 2026-09-19

**Authors:** Shenshi Jiang, Xiaoxiao Bu, Jane L. Whitelaw, Enbang Li

**Affiliations:** 1 School of Mathematics and Physics, University of Wollongong, Wollongong, NSW 2522, Australia; sj863@uowmail.edu.au; 2 School of Engineering, University of Wollongong, Wollongong, NSW 2522, Australia; xb376@uowmail.edu.au; 3 School of Medical, Indigenous and Health Sciences, University of Wollongong, Wollongong, NSW 2522, Australia; jwhitela@uow.edu.au

**Keywords:** whole-body vibration, ISO 2631-1, AS 2670.1, edge computing, MEMS accelerometer, frequency weighting, exposure metrics, metric-level telemetry, Internet of Things (IoT)

## Abstract

Timely feedback on whole-body vibration (WBV) exposure requires standardised metrics during measurement, yet many workflows record raw acceleration for offline processing, delaying feedback and increasing the data burden for bandwidth-constrained deployment. This paper presents a sensing node computing ISO 2631-1/AS 2670.1 exposure metrics on-device and transmitting metric records, not waveforms. The node combines an LIS2DH microelectromechanical systems (MEMS) accelerometer with an RP2040 microcontroller to calculate weighted root-mean-square (RMS) acceleration, daily exposure *A*(8), vibration dose value, maximum transient vibration value and crest factor. Algorithm-level verification showed that the implemented *W*_k_ filter reproduced the tabulated ISO 2631-1 response within 1.1% across 0.5–80 Hz. The node was compared under laboratory conditions with a CEM DT-178A datalogger whose 20 Hz recordings were reprocessed through a method-matched causal pipeline. Across ten trials per configuration under vertical excitation in the 6.3 Hz one-third-octave band, mean Z-axis *A*(8) differences were −4.5% (wired) and −7.9% (wireless). The datalogger’s 20 Hz sampling was included within the comparison chain, and the lower realised *W*_k_ gain was consistent with the direction and approximate magnitude of the observed offset. Metric-level telemetry reduced the sustained payload rate by up to four orders of magnitude at the summary cadence, supporting bandwidth-constrained uplinks.

## 1. Introduction

Whole-body vibration (WBV) is transmitted to the human body through a supporting surface such as a vehicle seat, cab floor or work platform. For operators of mobile machinery in mining, construction, agriculture and transport, WBV is a recognised occupational hazard [1,2,3,4,5,6,7], with the risk of low back pain and sciatica increasing with the magnitude and duration of exposure [8,9]. Such exposure is commonly assessed according to ISO 2631-1 and its Australian equivalent AS 2670.1, which apply axis-specific frequency weighting and time-based aggregation to the measured triaxial acceleration [10,11]. The resulting WBV metrics include weighted root-mean-square (RMS) acceleration ($a_{w}$), daily exposure *A*(8), vibration dose value (VDV), maximum transient vibration value (MTVV) and crest factor.

In WBV studies and field assessments, the standard-defined exposure metrics are commonly derived from recorded acceleration on a host computer rather than on the measuring device [12]. The post-acquisition workflow is well established but separates measurement from metric computation. *A*(8) and related metrics become available only after the recordings have been retrieved and processed, not while the measurement is in progress. Commercial human-vibration instruments remove the feedback delay by computing the metrics during measurement. However, proprietary firmware precludes independent review or adaptation of the weighting filters and metric calculations [13]. The cost of instrument-grade systems also constrains the number of units deployed simultaneously. In parallel, low-cost vibration sensing has advanced along two largely separate tracks, neither of which provides an auditable on-device implementation of the standard-defined WBV exposure metrics.

One line of low-cost sensing research has examined the measurement hardware itself. Microelectromechanical systems (MEMS) accelerometers for human vibration and low-frequency vibration measurement have been characterised for noise, bandwidth and amplitude linearity [14,15,16]. Related studies have also addressed metrological traceability and low-frequency accelerometer calibration [17,18,19,20,21]. Such studies establish that inexpensive sensors can provide usable acceleration measurements, with the standardised weighting and metric calculation performed during host-side post-processing. A parallel line of Internet of Things (IoT) research has emphasised edge processing and telemetry, where RMS, spectral or fault features are extracted on the node and transmitted as compact summaries [22,23,24,25,26,27,28,29]. On-node feature extraction and low-data-rate telemetry are therefore practical, but existing implementations primarily target machine diagnostics, not human WBV exposure metrics. Beyond on-node processing, edge-computing architectures can distribute computation through task offloading, for example, to roadside units and lightweight servers carried by unmanned aerial vehicles in vehicular systems [30,31]. Wireless WBV measurement has also been demonstrated, but earlier systems transmitted acquired acceleration over short-range links and computed the weighting and metrics on the host rather than at the sensing node [32].

These WBV exposure metrics should be computed on the sensing node in an auditable form, not reconstructed afterwards from stored acceleration or hidden within proprietary firmware. Such a node is implemented on off-the-shelf hardware: an RP2040 microcontroller and an LIS2DH MEMS accelerometer. The node applies the ISO 2631-1/AS 2670.1 *W*_d_/*W*_k_ weightings and computes *a*_w_, *A*(8), VDV, MTVV and crest factor on the device, and transmits only the resulting exposure-metric records over a short-range wireless link.

The node is evaluated under laboratory conditions using a CEM DT-178A triaxial datalogger as a practical comparison instrument. Recordings from the datalogger are reprocessed through a method-matched causal pipeline. The evaluation covers two configurations: a wired sensing-and-computation core and an end-to-end wireless prototype. Both are tested under controlled vertical excitation in the one-third-octave band centred at 6.3 Hz. The selected condition lies within the same *W*_k_-sensitive low-frequency region as the dominant component observed near 8 Hz during prior field measurements. The 6.3 Hz band also reduces the sampling-rate-induced weighting distortion in the 20 Hz reference-processing chain relative to an 8 Hz condition. The laboratory comparison evaluates agreement between the two measurement chains at 6.3 Hz.

The principal contribution is an inspectable node-level implementation of ISO 2631-1 exposure metrics using low-cost hardware. A secondary contribution is the demonstration of metric-level telemetry as a communication-efficient alternative to raw vibration streaming. The accompanying laboratory comparison treats the practical datalogger as a component of the comparison chain rather than as an absolute reference.

## 2. Standards-Based Requirements for WBV Exposure Monitoring

ISO 2631-1:1997 (with Amendment 1:2010) and AS 2670.1—2001 (Reconfirmed 2016, with Amendment 1:2013) share a common framework for seated WBV health assessments and are referred to jointly as the standard [10,11]. This section restates the provisions that govern such assessment and translates each into a node-level requirement. Table 1 summarises the resulting standard-to-node mapping and provides the design basis for Section 3.

### 2.1. Measurement Configuration and Frequency Range

For health assessment of a seated person, the standard places the accelerometer at the seat surface beneath the ischial tuberosities, where the measured motion represents the vibration transmitted to the body. The transducer axes are orthogonal, with up to 15° deviation permitted where exact alignment is impractical. Seated WBV health-risk assessment covers the 0.5–80 Hz health band. Accordingly, the node design requires a transducer and sampling system capable of resolving this band with adequate frequency response and dynamic range.

### 2.2. Processing Chain as a Sequence of Design Requirements

The standard sets an ordered processing sequence: band-limited signal conditioning, per-axis frequency weighting, the basic weighted RMS, a crest-factor sufficiency check, additional metrics where transient peaks may be missed, and daily-exposure calculation against the health guidance caution zone (HGCZ). As an implementation specification, this sequence maps onto causal operations on the live sample stream, from which every metric is derived. The standard also allows separate periods of varying exposure to be analysed and combined, which the node supports by keeping accumulators per measurement segment.

### 2.3. Frequency Weighting and Multiplying Factors

Frequency weighting accounts for the variation in human response with vibration frequency and direction. Annex A defines each weighting as an analogue transfer function, $H(s)=H_{h}(s)\;H_{l}(s)\;H_{t}(s)\;H_{s}(s)$, factoring into band-limiting, acceleration–velocity transition and weighting-specific shaping sections. The vertical weighting *W*_k_ adds an upward-step shaping component absent from the horizontal *W*_d_. For seated health assessments, the standard assigns *W*_d_ to the x- and y-axes, with *k_x_* = *k_y_* = 1.4, and *W*_k_ to the z-axis, with *k_z_* = 1.0. The weighting filters act on the acceleration time history. The multiplying factors are applied later, during exposure evaluation. The node requirement is to discretise the Annex A transfer functions into causal IIR filters, applied sample-by-sample per axis, with the factors kept separate. Both the weighted and the k-applied quantities then remain available. The present evaluation focuses on the Z-axis under vertical excitation. With *k_z_* = 1.0, the weighted and k-applied RMS values coincide.

### 2.4. Exposure Metrics

The node derives the five exposure metrics from running accumulators and a 1 s circular buffer. The weighted RMS acceleration, $a_{w}={\left[\frac{1}{T}\int_{0}^{T}a_{w}^{2}(t)\;dt\right]}^{1/2}$, is calculated from a running sum of squared weighted samples and the corresponding sample count for each axis. The crest factor, $CF=\frac{a_{\mathrm{peak}}}{a_{w}}$, the ratio of peak to RMS weighted acceleration, is calculated from the running absolute peak and the weighted RMS. CF ≤ 9 indicates vibration adequately described by RMS, and CF > 9 warrants additional metrics. The MTVV is the maximum of a short-time running RMS, $MTVV=max[a_{w}(t_{0})]$ over a recommended *τ* = 1 s window, capturing short events the full-duration RMS would mask. The $VDV=[\int a_{w}^{4}(t)dt{]}^{1/4}$ is more peak-sensitive and is accumulated as a running fourth-power sum, with ${VDV}_{total}={\left(\sum_{i}{VDV}_{i}^{4}\right)}^{1/4}$ across periods. The daily exposure normalises the weighted magnitude to $T_{0}=28,800\;s$, $A_{l}\left(8\right)=k_{l}\sqrt{\frac{1}{T_{0}}\sum_{i}a_{wli}^{2}T_{i}}$ with $A(8{)}_{max}=max\left[A_{x}(8),A_{y}(8),A_{z}(8)\right]$. Segments combine by the standard’s energy and fourth-power rules.

### 2.5. Reporting and Interpretation

Results are interpreted against the HGCZ, which the standard treats as duration-dependent guidance rather than a single-threshold boundary. Interpretation requires both the weighted magnitudes and the corresponding exposure durations, which the per segment accumulators retain.

Table 1 collects the provisions and the corresponding node implementation. At node level, the requirements are realised as a transducer with adequate frequency response, causal per-axis weighting filters, a set of running accumulators and configured guidance boundaries.

## 3. Edge-Computing WBV Sensor Platform

**Figure 1:**
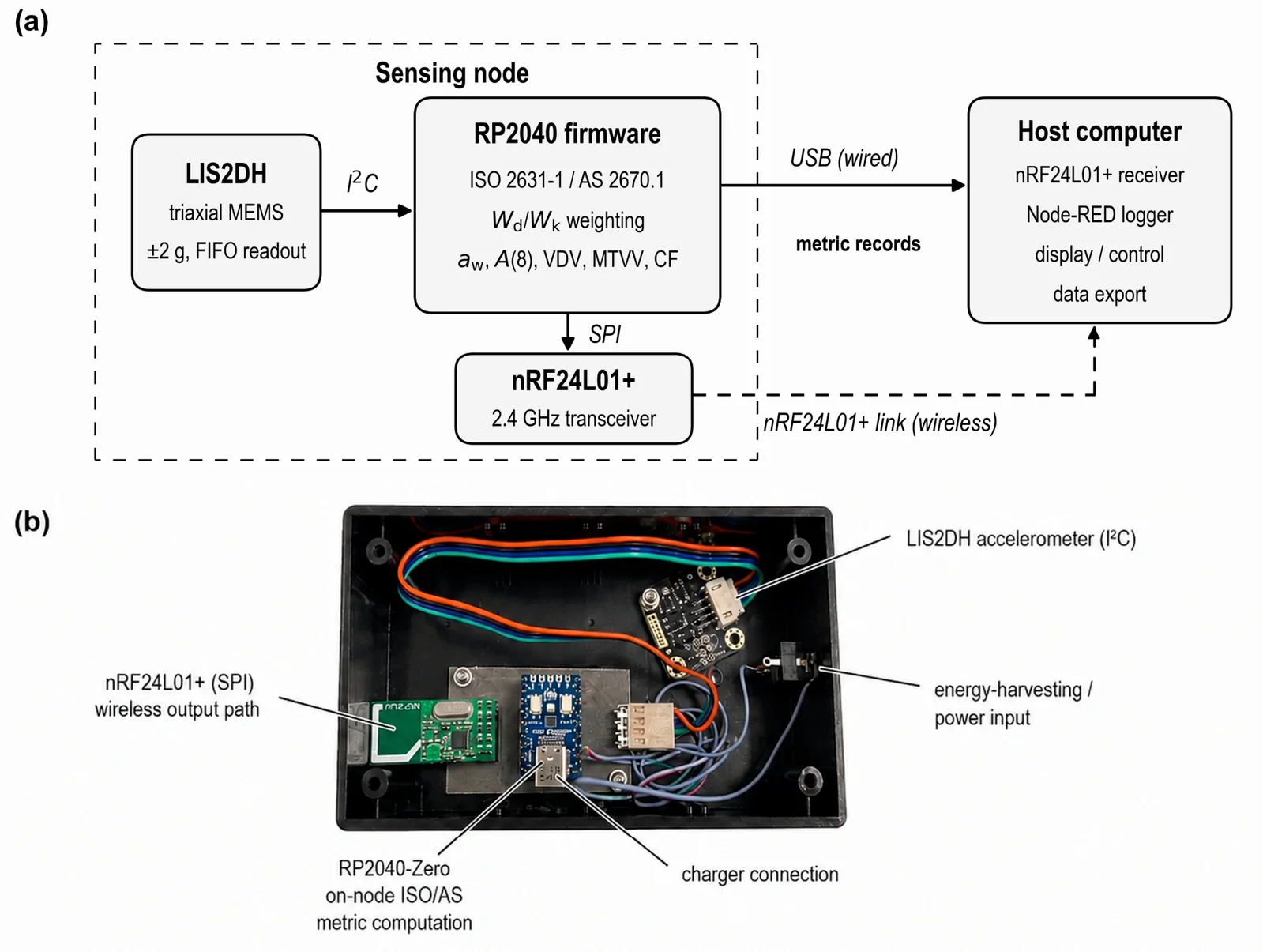
Edge-computing whole-body vibration (WBV) sensor platform: (**a**) system architecture and (**b**) prototype sensing node hardware.

The requirements of Section 2 were implemented as a common sensing-and-computation core that converts the live triaxial acceleration stream into standard-defined WBV exposure records. The same embedded computation serves two output paths: USB CDC for the wired core and an nRF24L01+ radio link for the wireless prototype. The mounting arrangements used for evaluation are described in Section 4. Figure 1 summarises the node architecture and data flow.

### 3.1. Hardware and Output Paths

The node was implemented with an RP2040-Zero microcontroller board (Waveshare, Shenzhen, China) and an LIS2DH triaxial MEMS accelerometer module (DFRobot, Shanghai, China). The accelerometer was configured at ±2 g full scale and read over I^2^C using its internal FIFO. The embedded weighting filters and time-dependent metric calculations used a nominal 1600 Hz firmware time base. Metric records, not raw acceleration waveforms, are delivered to the host PC either over USB CDC or through an nRF24L01+ 2.4 GHz transceiver (nRF24L01+ IC: Nordic Semiconductor, Trondheim, Norway), driven over SPI, to a matching receiver. The Type-C port provides the USB CDC output and charging, and a separate enclosure connector accepts a battery or harvester supply. The receiver, a second RP2040 with an nRF24L01+, decodes the fixed-point payload fields, forwards the records to the host PC over USB CDC as text and appends sequence-based frame-loss counts to the output. The receiver performs no weighting or metric computation. Low-power wide-area links are a separate deployment path.

### 3.2. Embedded Processing Chain

The firmware applies the standard’s processing sequence to the live sample stream. Before each measurement, a static offset calibration estimates fixed per-axis offsets during a short stationary interval. The offsets are subtracted throughout the active segment, and dynamic baseline tracking is disabled to keep the baseline fixed during the measurement. Each corrected sample is then passed through the axis-specific Annex A weighting filters, implemented as causal IIR sections, *W*_d_ for the horizontal axes and *W*_k_ for the vertical axis, to give the instantaneous weighted acceleration [33].

From the weighted stream, the node updates running accumulators without storing the waveform. Per axis and segment, the node maintains a running sum of squared weighted samples (Σ*a*_w_^2^) with a sample count for *a*_w_. A running maximum of the absolute weighted sample supports the crest-factor calculation. A 1600-sample circular buffer of squared weighted samples implements the nominal 1 s window and retains the maximum short-time RMS for MTVV. A running sum of fourth powers (Σ*a*_w_^4^) accumulates VDV. At segment close, the accumulators are converted into *a*_w_, VDV, MTVV and crest factor. The multiplying factors and segment duration then enter the *A*(8) calculation, and the per segment weighted energy and duration contribute to the cumulative *A*(8). All selected metrics derive from accumulators. The memory footprint remains constant regardless of segment length. The RP2040-Zero executed the processing chain continuously throughout the evaluated trials. Exposure durations are derived from the sample count using the nominal 1600 Hz firmware time base. The per-sample filtering runs in single precision, and every accumulator is kept in double precision.

### 3.3. Metric Records and Host Interface

The node emits metric records at three cadences: a 1 s diagnostic record for live monitoring, a 60 s summary record, and a segment-level record carrying the final *a*_w_, *A*(8), VDV, MTVV and crest factor with the segment duration. In the wireless frames, the metric fields are carried as fixed-point integers with a resolution of 0.001 m/s^2^. The fixed-point step is equivalent to at most 1.3% of the smallest per segment *A*(8) and less than 0.15% of the weighted-RMS amplitudes in the laboratory comparison. The wired path outputs full-precision values over USB CDC. The waveform is not preserved: once the metrics are formed, the acceleration record is no longer available for re-analysis under a different weighting or for inspecting individual transients. The reduction in data burden relative to raw acceleration streaming is quantified in Section 5.3.

The same host-side software layer receives, logs and displays the records in both output paths. The host also issues measurement-control commands to the node: request the current exposure metrics, end a segment, reset the daily *A*(8), or export the on-node log. When the operator requests the current metric values, the dashboard displays the returned values immediately and flags metrics that exceed configured warning or alarm thresholds. Exposure status is available during the measurement, not only after download. The host layer is built from standard Node-RED 4.1.7 components.

## 4. Verification and Evaluation Methods

The evaluation is organised at three levels. Algorithm-level verification checks the embedded weighting and metric calculations without involving the physical sensing chain. The wired evaluation covers the sensing-and-computation core under side-by-side plate mounting and compares the node with a practical datalogger processed through a method-matched pipeline. The wireless evaluation covers the complete prototype, including the radio link, enclosure and stacked mounting. The remaining method subsections define the reference-processing pipeline, apparatus, excitation, comparison method and prespecified engineering criteria.

### 4.1. Algorithm-Level and On-Target Verification of the Embedded Weighting Filters

The embedded weighting and metric calculations were verified before the physical comparison with the datalogger. The ISO 2631-1 Annex A weighting filters implemented on the node were reconstructed in Python 3.11.9. Each weighting filter was implemented as a series of causal IIR sections corresponding to the component transfer functions specified in Annex A. Sinusoidal and transient test signals were used to compare the reconstructed weighting responses with the ISO 2631-1:1997 Table 3 values and the computed metric values with analytical references. The reconstructed *W*_k_ magnitude matched the tabulated values to within 1.1% across 0.5–80 Hz, and the single-precision contribution to the weighted RMS remained below 0.21%.

**Figure 2:**
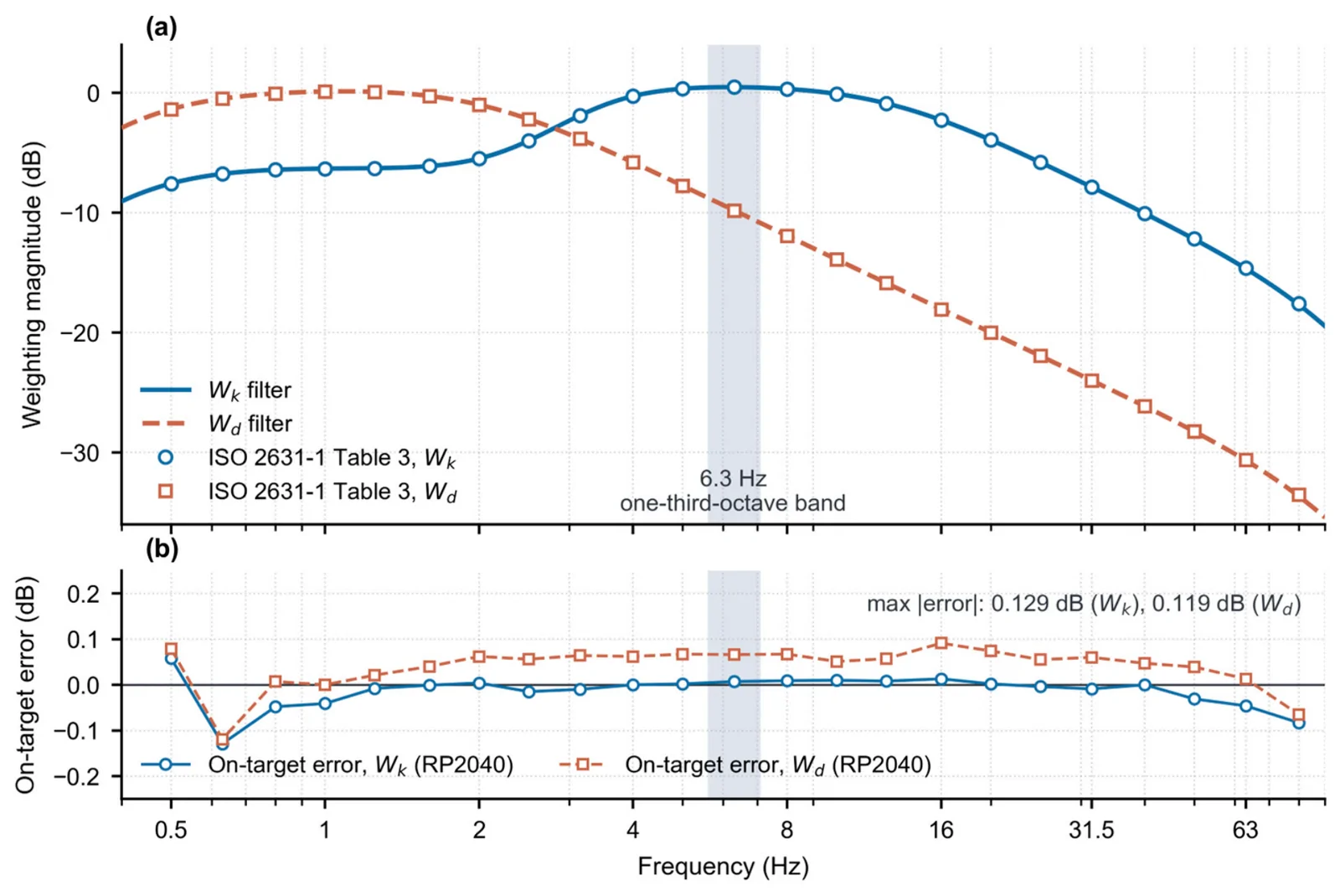
RP2040 verification of the *W*_k_ and *W*_d_ weighting filters against ISO 2631-1:1997 Table 3: (**a**) magnitude responses; (**b**) on-target errors. The shaded region marks the 6.3 Hz one-third-octave band.

The same weighting header used in the measurement firmware was then checked on the target RP2040. At the nominal 1600 Hz firmware time base, the compiled *W*_k_ and *W*_d_ weighting filters matched the tabulated magnitudes across 0.5–80 Hz, with maximum absolute errors of 0.129 dB and 0.119 dB, respectively (Figure 2). At the 6.3 Hz band centre, the *W*_k_ error was 0.08%; at the adjacent 8 Hz reference point, it was 0.10%. These checks support the weighting implementation used in the node firmware.

### 4.2. Reference-Processing Pipeline and Its Sampling-Rate Limitation

The CEM DT-178A triaxial datalogger (Shenzhen Everbest Machinery Industry Co., Ltd., Shenzhen, China) was used as a practical comparison instrument, not as an absolute reference. During the 6.3 Hz laboratory comparison, the datalogger provided an independent measurement chain, with both systems mounted on the same vibration plate and recording concurrently. The weighting implementation over 0.5–80 Hz was verified separately through the algorithm-level and on-target checks described in Section 4.1. Native 20 Hz recordings from the datalogger were reprocessed offline in Python without resampling. The reprocessing applied the same metric definitions and causal processing sequence as the node. The weighting filters were discretised with the bilinear transform. The filters applied to the datalogger records used the native 20 Hz sampling rate. The node filters used the nominal 1600 Hz firmware time base. The two chains were matched in method rather than in realised frequency response. Frequency weighting was applied by single-pass causal IIR filtering, not by zero-phase filtering such as filtfilt. The offline reference, like the embedded node, used no future-sample information. Offset removal followed the node offset-estimation procedure: the mean of the first 2 s of the stationary interval was subtracted from each record, and the same interval was excluded from metric calculation as a warm-up skip. No spike removal was applied in the reference processing, avoiding any offline cleaning step unavailable to the real-time node.

For both configurations, datalogger records were cropped to the segment windows reported by the node. Each window was reconstructed as the segment end time minus the reported exposure duration. For the wired configuration, the end time was taken from the node-reported UTC timestamp, with the host-received timestamp as a fallback. For the wireless configuration, the end time was taken from the host timestamp of the final segment-level record. When a test contained more than one segment, the reference metrics were computed separately for each reconstructed window and then combined by the same energy-based RMS/*A*(8) and fourth-power VDV rules applied to the node outputs. The 20 Hz sampling rate gives a 10 Hz Nyquist frequency, leaving limited margin above the dominant 6.3 Hz band. Near the 10 Hz Nyquist frequency, bilinear-transform warping causes the realised causal *W*_k_ response at 20 Hz to fall increasingly below the response of the nominal 1600 Hz node-processing implementation. One-third-octave analysis served only to characterise the excitation spectrum and identify the dominant 6.3 Hz band. The primary node-to-datalogger comparison used the time-domain exposure metrics. Neither chain had a traceable amplitude calibration. The comparison therefore establishes agreement between two method-matched chains, not absolute accuracy.

### 4.3. Experimental Apparatus and Excitation

**Figure 3:**
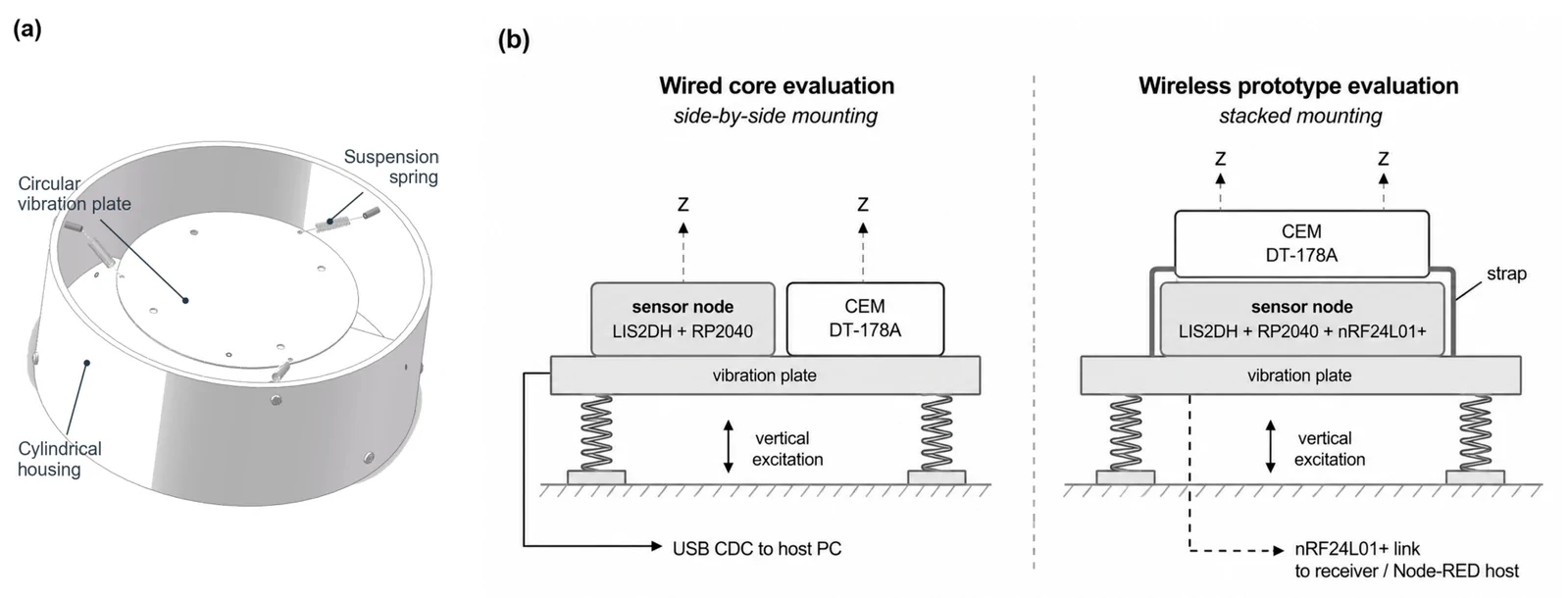
Experimental apparatus: (**a**) vibration test rig and (**b**) mounting arrangements for the wired (side-by-side) and wireless (stacked) evaluations.

**Figure 4:**
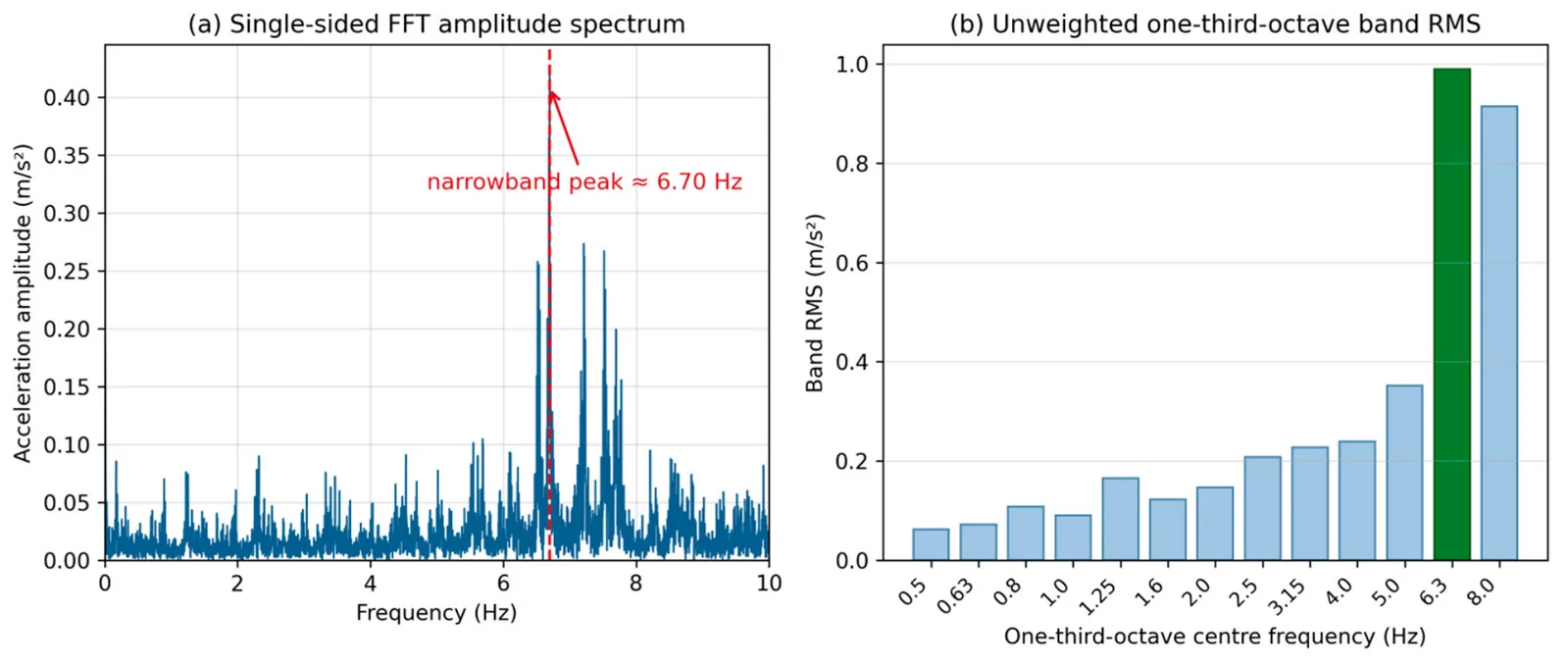
(**a**) Single-sided FFT amplitude spectrum and (**b**) unweighted one-third-octave band RMS for a representative datalogger recording. In (**b**), the dominant 6.3 Hz band is shown in green, and the remaining bands are shown in blue.

Vibration was generated by a laboratory test rig with a sprung circular vibration plate inside a cylindrical housing. The plate served as the common mounting surface for the node and the datalogger (Figure 3). A Thurlby Thandar Instruments TG120 function generator (Huntingdon, UK) drove the test rig with a sinusoidal input. The analogue frequency dial provided only a nominal setting. The effective excitation was characterised from the recorded acceleration spectra. One-third-octave analysis placed the dominant vertical component in the band centred at 6.3 Hz in all twenty trials. The 6.3 Hz band exceeded the adjacent 8 Hz band by a factor of 1.01–1.41. The relatively small margin indicated spectral content extending toward the band boundary. The one-third-octave band RMS values used for band identification were computed offline from the unweighted acceleration records exported by the datalogger. The *W*_k_ weighting described in Section 4.2 was applied when calculating the datalogger-derived Z-axis exposure metrics. The 20 Hz *W*_k_ discretisation was therefore used only in the exposure-metric calculation. Figure 4 shows a representative recording, with the largest narrowband peak at approximately 6.7 Hz. The narrowband peak is specific to the laboratory apparatus.

**Figure 5:**
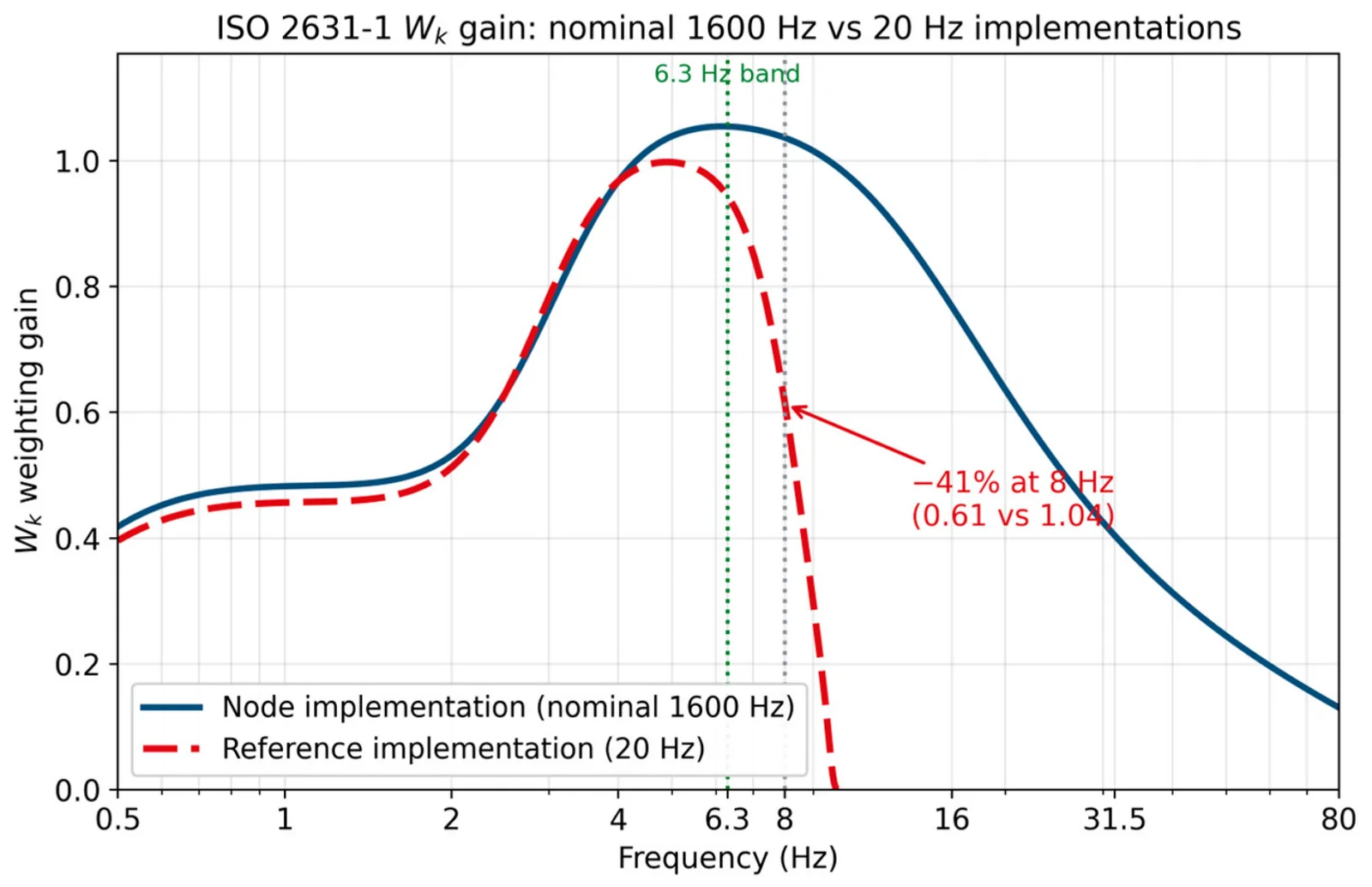
Causal *W*_k_ gain for the nominal 1600 Hz node-processing implementation and the 20 Hz reference-processing chain. The vertical lines mark 6.3 Hz (green) and the 8 Hz reference point (grey).

The 6.3 Hz band lies in the 4–8 Hz region of greatest vertical sensitivity under *W*_k_. Prior mining-equipment measurements motivating the test condition showed a dominant component near 8 Hz, within the same region. Relative to the nominal 1600 Hz node-processing implementation, the realised causal *W*_k_ magnitude at 20 Hz is approximately 41% lower at 8 Hz and 10.5% lower at 6.3 Hz (Figure 5). The test condition was therefore set at 6.3 Hz. The 6.3 Hz condition also reduced the sampling-rate-induced weighting distortion in the 20 Hz reference-processing chain relative to an 8 Hz condition.

Each measurement window was approximately five minutes with the excitation held constant. The node-side weighted amplitude ranged from approximately 0.12 to 0.23 m/s^2^ in the wired trials and from approximately 0.8 to 1.5 m/s^2^ in the wireless trials. These amplitude ranges describe the two test sessions and do not constitute a matched-amplitude comparison. The node and datalogger were mounted on the same vibration plate with both Z-axes vertical. The wired core used side-by-side mounting. The wireless prototype used stacked mounting, with the datalogger secured above the node. Both evaluations followed the same trial sequence. Before each trial, the datalogger recording was started, a stationary interval of 10–15 s established the offset baselines of both chains, the on-node accumulators were reset and a new measurement segment was begun. After each trial, the excitation was switched off and the segment was closed to produce the final segment-level record. The datalogger recording was stopped last. The stop order ensured that the reference record covered the full node segment.

### 4.4. Wired Core Evaluation Protocol

The wired configuration evaluates the sensing-and-computation core (LIS2DH + RP2040) in isolation from the wireless link. Ten trials were conducted while per-segment records were streamed from the node to the host PC over USB CDC.

### 4.5. Wireless End-to-End Evaluation Protocol

The wireless configuration evaluates the complete prototype, including on-node computation and the nRF24L01+ radio link. Metric records were transmitted from the node through the receiver node to the Node-RED host, and reception of the diagnostic stream was confirmed before each trial. Ten trials were conducted in a separate session from the wired trials.

### 4.6. Comparison Method and Prespecified Engineering Criteria

The primary comparison quantities are the Z-axis *W*_k_-weighted RMS acceleration *k_z_*·*a*_w,*z*_ (*k_z_* = 1.0) and the corresponding *A_z_*(8). VDV and crest factor are used as supporting indicators. The node computed MTVV, which was not treated as a separate comparison endpoint under the steady laboratory excitation. The *A*(8) values are computed from the bench segment durations under the standard’s eight-hour normalisation. The values serve as comparison quantities between the two chains, not as estimates of occupational daily exposure. The node implements axis-specific processing for all three axes. The present physical comparison focused on the Z-axis because the apparatus provided controlled vertical excitation only and the stacked wireless mounting lacked a controlled common horizontal reference.

The difference is defined as 100 × (CEM − node)/node. A negative value indicates that the reference value is lower than the corresponding node value. Agreement is summarised by the mean difference, the standard deviation (SD) of the per-trial differences, the Pearson correlation and Bland–Altman limits of agreement. Two-sided 95% confidence intervals for the mean percentage differences were calculated using Student’s *t* distribution with *n* − 1 degrees of freedom. Confidence intervals for Pearson correlation coefficients were obtained using Fisher’s *z* transformation. Bland–Altman bias and 95% limits of agreement were calculated from the paired absolute differences, defined as CEM minus node, using the mean difference ± 1.96 SD. The grand-mean amplitude used to contextualise the absolute bias and limits of agreement was calculated as the mean of all paired node and datalogger weighted-RMS values. The wired trials covered a limited amplitude range. The correlation therefore serves as a supporting indicator. Bland–Altman analysis is treated as the primary agreement assessment.

For the wired core, the engineering criteria were set a priori at |mean difference| < 15%, SD < 10%, *r* > 0.90 and *n* ≥ 10. The mean-difference bound was chosen as a practical agreement limit that allowed for the expected attenuation of the 20 Hz reference chain under the planned 6.3 Hz test condition. The criteria were used only for the initial laboratory comparison and do not represent standards-based conformance thresholds. The numerical engineering criteria were applied to the point estimates, and the sample-size criterion was assessed directly. Confidence intervals were reported to indicate sampling uncertainty and did not determine whether the engineering criteria were met. The engineering criteria apply only to the primary weighted-RMS and *A*(8) comparison. The supporting indicators are reported without acceptance thresholds. The wireless evaluation is reported descriptively using the same quantities and does not constitute an isolated acceptance test of the radio link.

## 5. Results

### 5.1. Wired Core Evaluation

**Figure 6:**
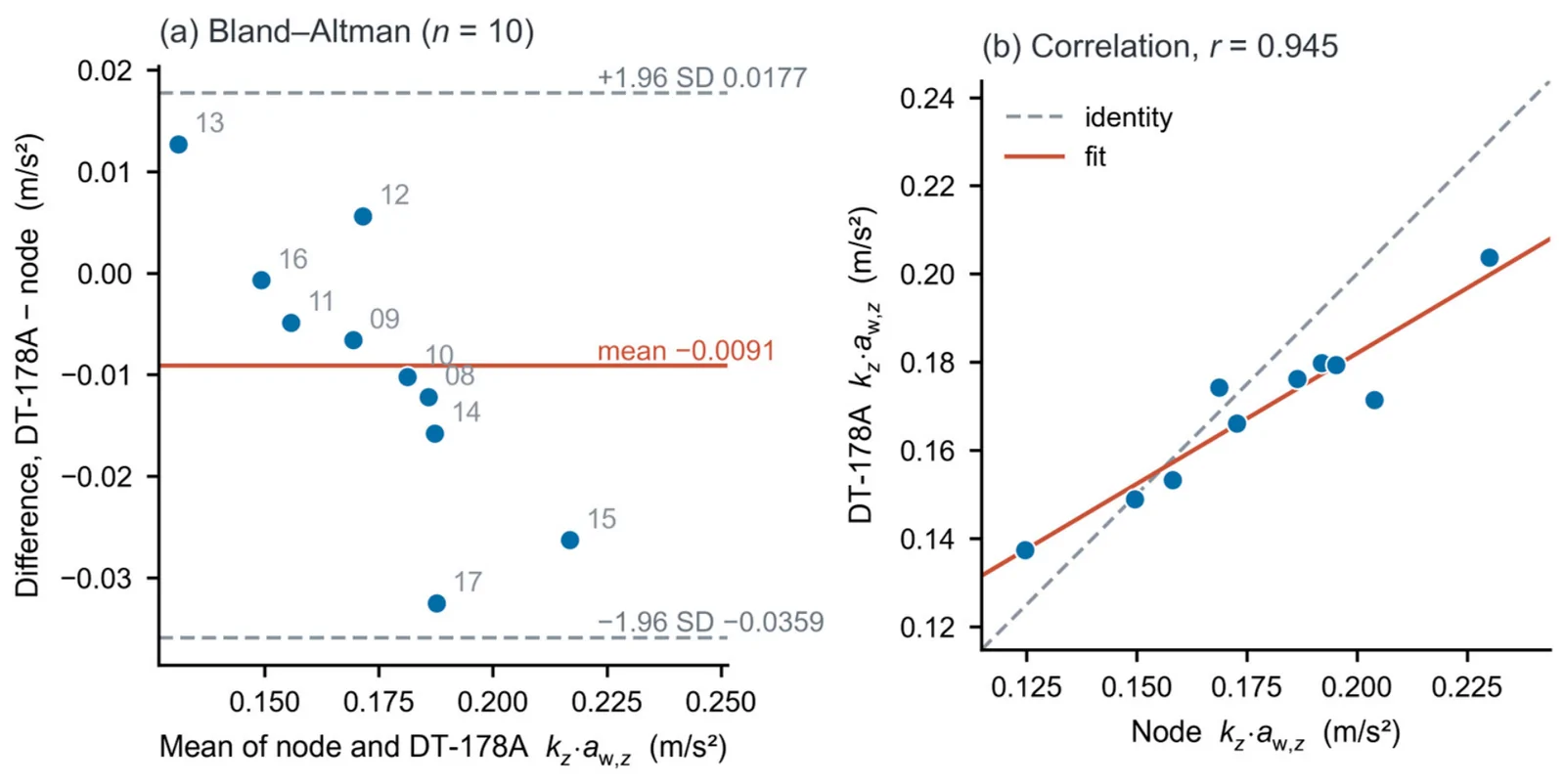
Wired core evaluation against the reprocessed reference (Z-axis, *n* = 10): (**a**) Bland–Altman plot based on paired absolute differences and (**b**) correlation plot (*r* = 0.945, 95% CI 0.779–0.987). Numbers in (**a**) denote the trial identifiers listed in Table 2.

The wired core met the prespecified mean-difference, standard-deviation and sample-size criteria for the primary quantities. Table 2 lists the per-trial results. The mean *A_z_*(8) difference was −4.53% (SD 7.78%, 95% CI −10.10% to 1.04%), and the corresponding Z-axis *k_z_*·*a*_w,*z*_ difference was −4.10%. For the primary weighted-RMS comparison, the Pearson correlation point estimate of 0.945 exceeded the 0.90 criterion. Bland–Altman analysis of the paired absolute differences gave a mean bias of −0.009 m/s^2^ and 95% limits of agreement from −0.036 to +0.018 m/s^2^, at a grand-mean amplitude of 0.173 m/s^2^ (Figure 6). The per-trial *A*(8) differences spanned [−17.4%, +10.2%]. The mean VDV difference was +10.30%. The node crest factor remained below nine in every trial. The reprocessed CEM crest factor exceeded nine in three trials (Table 2). The reference-derived RMS comparisons for the three CEM CF > 9 trials are interpreted with additional caution, and VDV is retained as a peak-sensitive supporting indicator.

### 5.2. Wireless End-to-End Evaluation

For the ten wireless trials, the mean *A_z_*(8) difference was −7.86% (SD 2.77%, 95% CI −9.84% to −5.88%), with a range of [−10.86%, −1.62%]. The mean *k_z_*·*a*_w,*z*_ difference was −7.67%, and the mean VDV difference was −8.31%. The Pearson correlation across the ten wireless pairs was 0.994. Bland–Altman analysis of the paired absolute differences gave a mean bias of −0.109 m/s^2^ and 95% limits of agreement from −0.195 to −0.023 m/s^2^, at a grand-mean amplitude of 1.303 m/s^2^. Nine of the ten trials clustered at *k_z_*·*a*_w,*z*_ ≈ 1.25–1.52 m/s^2^. Excluding the lowest-amplitude trial reduced the correlation from 0.994 to 0.940. Both correlation estimates are reported without acceptance thresholds. The node Z-axis crest factor remained below nine in all trials. The wireless VDV difference had the same negative sign as the RMS and *A*(8) differences, contrasting with the positive wired VDV difference. The sign contrast is examined in Section 6.2.

Across the ten trials, 10–17% of the 1 s diagnostic frames were lost over the nRF24L01+ link. The segment-level records used for the comparison were received in every trial. On-node metric accumulation prevented diagnostic-frame loss from affecting the per-segment values. For *A*(8), the wireless prototype showed a numerically larger absolute mean percentage difference and a smaller trial-to-trial spread than the wired core. Table 3 summarises the two configurations, including the complete set of confidence intervals.

### 5.3. Telemetry Data-Burden Quantification

Table 4 compares the payload-level sustained data rate of raw acceleration streaming with the three metric-record cadences. Raw three-axis streaming referenced to the nominal 1600 Hz firmware time base, with two bytes per axis, requires approximately 9.6 kB/s. The metric records contain tens of payload bytes and are emitted once per second, once per minute or once per segment. At the 60 s summary cadence, the sustained rate falls roughly four orders of magnitude below raw streaming. The selected standard-defined exposure metrics are retained in the transmitted records.

## 6. Discussion

### 6.1. Scope of the Evaluation

The wired and wireless configurations require separate interpretation. The wired core (Section 5.1) exercises the MEMS sensing chain and on-node computation under side-by-side mounting with no wireless link. The wireless prototype (Section 5.2) covers the complete end-to-end behaviour, including the radio link and the stacked mounting. The algorithm-level verification (Section 4.1) isolates the embedded arithmetic from any physical effect. The configurations also differed in mounting, link, session and excitation amplitude. The cross-configuration comparison is therefore interpretive rather than a controlled decomposition. The analysis identifies plausible sources of the difference without quantitatively partitioning their individual contributions. The reported statistics quantify agreement between the two measurement chains under the evaluated conditions, not absolute measurement accuracy.

### 6.2. Sources of the Observed Difference

The common negative *A_z_*(8) offset is consistent with attenuation in the 20 Hz reference-processing chain. At the 6.3 Hz band centre, the realised causal *W*_k_ magnitude at 20 Hz is approximately 10.5% lower than the corresponding magnitude of the nominal 1600 Hz node-processing implementation, with the difference increasing toward the 8 Hz band edge (Figure 5). The digital weighting difference provides a plausible explanation for the direction and approximate magnitude of the observed offset. The 10.5% value characterises the difference between the two digital *W*_k_ implementations alone. The value is not a prediction of the complete node-to-datalogger difference, which also reflects the physical sensing chains, mounting arrangements, excitation spectra and session-specific conditions.

The configurations differed in the balance between mean offset and trial-to-trial spread. The observed pattern may partly reflect a mounting-transfer effect. A stacked arrangement could introduce a more repeatable transfer path, shifting the mean and reducing the trial-to-trial variance. A side-by-side arrangement may be more placement-sensitive, consistent with the smaller mean and wider spread of the wired trials. The narrow range of wireless percentage differences is consistent with a systematic rather than intermittent contribution.

The wireless trials were also conducted at a higher excitation amplitude than the wired trials (Section 4.3). The higher excitation amplitude may have improved the signal-to-noise ratio and contributed to the smaller spread. The VDV sign reversal between configurations (positive wired, negative wireless) is consistent with this interpretation. At the higher wireless amplitude, the fourth-power integral may have been dominated by the sustained sinusoid, for which the 20 Hz reference-processing chain yields a lower realised *W*_k_ magnitude. At the lower wired amplitude, individual peaks in the reference chain may have had greater relative weight and increased the reference-derived VDV. The concurrent experimental changes prevent separation of the individual contributions.

### 6.3. Low-Data-Rate Telemetry for IoT Monitoring

Metric-level telemetry changes the role of the wireless link from waveform transport to exposure reporting. The data-burden comparison in Table 4 shows that metric-level records require a substantially lower sustained data rate than raw acceleration streaming. The evaluated prototype demonstrates low-data-rate exposure reporting over the short-range nRF24L01+ link. The quantified payload rates provide a basis for future evaluation with LoRa-class low-power wide-area uplinks [23,25]. The three record cadences could support future duty-cycle control, with segment records for low-rate exposure logging and diagnostic records for periods requiring closer inspection.

On-node accumulation separated exposure estimation from continuous diagnostic delivery. The 1 s diagnostic-frame losses did not alter the final segment metrics, with accumulation retained on the sensing node and the segment-level records received in every trial. Transmitting exposure metrics rather than the waveform removes the option of later reprocessing under alternative weightings or transient-level inspection. Long-range deployment will require mechanisms to ensure segment-level delivery and to quantify protocol overhead, retransmission and energy cost.

### 6.4. Energy Requirements for Remote Deployment

Remote deployment requires low-power operation to limit battery servicing [34]. Quantifying node-level energy consumption across sampling, computation, transmission, storage and power management lies outside the present evaluation. The evaluated sensing-and-computation core provides the basis for a dedicated energy assessment.

### 6.5. Limitations

The physical comparison covered the Z-axis *W*_k_ response under controlled vertical excitation in a single one-third-octave band centred at 6.3 Hz. Characterisation of the complete sensing chain requires a physical sweep across the *W*_k_-sensitive region, including 4, 6.3 and 8 Hz, together with traceable amplitude calibration. Each configuration comprised ten trials. The confidence intervals quantify uncertainty in the estimates obtained from the evaluated trials and should not be interpreted as performance bounds for broader deployment conditions. The limits of agreement are point estimates and should be interpreted as indicative.

The X/Y weighting (*W*_d_, *k* = 1.4) is implemented on the node but has not yet undergone a controlled horizontal-axis evaluation. The reported evaluation does not include broadband, multi-axis field exposure records. The compiled *W*_k_ and *W*_d_ filters were verified on the target RP2040 across the 0.5–80 Hz health band at the nominal firmware time base. The node computes axis-specific exposure metrics for X, Y and Z using the prescribed *W*_d_/*W*_k_ weightings, with the multiplying factors applied during exposure evaluation. This per-axis output is consistent with the standard’s requirement that health be assessed independently along each axis. The processing chain therefore supports exposure assessment across the 0.5–80 Hz health band and under multi-axis exposure. Broader physical characterisation should include controlled tests at additional frequencies and under horizontal or simultaneous multi-axis excitation to quantify the realised response of the transducer and mounting.

Post hoc inspection of logged sample counts indicated unit- and session-dependent deviations from the nominal 1600 Hz firmware time base. Some minute records also contained lower sample counts. The lower-count records were not accompanied by logged FIFO-overrun events during the twenty formal trials. Both chains were windowed using the same node-reported durations (Section 4.2), preserving nominal window alignment for the relative comparison under steady excitation. A deviation from the nominal time base shifts the physical-frequency mapping of the *W*_k_ and *W*_d_ weighting responses and can alter the weighted acceleration used in the WBV metric calculations. At the adjacent one-third-octave band centre frequencies of 5 and 8 Hz, the tabulated *W*_k_ values are within 1.8% of the 6.3 Hz value. The shared node-reported duration does not enter the relative *A*(8) and VDV differences. For standalone node calculations, a deviation from the nominal time base affects the conversion from sample count to time, thereby affecting the reported exposure duration, *A*(8), VDV and the effective duration of the nominal 1 s MTVV window.

Only the short-range nRF24L01+ link was evaluated. The on-node guidance-band classification ran throughout the trials, and transmitted records carried the resulting risk-band flags. The present comparison evaluates the underlying metric values, not the classification outcomes as a separate endpoint.

## 7. Conclusions

The results show that the selected ISO 2631-1/AS 2670.1 WBV exposure-metric chain can be implemented on a low-cost RP2040/LIS2DH sensing node without retaining the raw acceleration waveform. The standard’s weighting and accumulation requirements were realised as causal per-axis filters and running accumulators. Algorithm-level reconstruction reproduced the tabulated *W*_k_ response to within 1.1% across 0.5–80 Hz. On-target checks showed maximum deviations below 0.13 dB for the compiled *W*_d_/*W*_k_ weighting filters. Metric-level telemetry retained the selected exposure metrics and reduced the sustained payload data rate by up to four orders of magnitude at the summary cadence.

Under laboratory excitation in the one-third-octave band centred at 6.3 Hz, the mean relative *A_z_*(8) differences were within 8% for both configurations, with the datalogger reporting lower values on average. The CEM DT-178A served as a practical comparator rather than an absolute reference, and the datalogger’s native 20 Hz sampling rate was retained in the comparison chain. The physical evidence applies to the vertical axis and the selected one-third-octave band. Within this scope, the results support the platform architecture for on-device WBV metric computation and low-data-rate exposure monitoring at operator contact surfaces on machinery. Further work will extend the evaluation through traceable amplitude calibration, multi-frequency and multi-axis testing, and a complete energy budget for remote deployment.

## Figures and Tables

**Table 1:** ISO 2631-1/AS 2670.1 provisions for seated whole-body vibration (WBV) health assessment and the corresponding node implementation.

Standard Provision	Node Implementation
Triaxial measurement over the 0.5–80 Hz health band	Triaxial LIS2DH acquisition through the internal FIFO, with firmware weighting and metric calculations referenced to a nominal 1600 Hz time base. The implementation was designed to support measurement over the 0.5–80 Hz health band
*W*_d_ on x/y and *W*_k_ on z, applied before any metric	Annex A transfer functions ^a^ as bilinear-discretised causal IIR filters, per sample and per axis
Multiplying factors *k_x_* = *k_y_* = 1.4, *k_z_* = 1.0	*k_x_* = *k_y_* = 1.4, *k_z_* = 1.0 as firmware constants, weighted and k-applied values both retained
Weighted RMS *a*_w_ over duration *T*	*a*_w_ = (Σ*w*^2^/*N*)^1/2^ with *T* = *N*/*f*_s_ ^b^
Crest-factor sufficiency test, CF > 9	Running weighted peak, with the CF = 9 decision flagged in each record
MTVV, 1 s running RMS, report if MTVV/*a*_w_ > 1.5	Sliding RMS at *τ* = 1 s, maximum retained in each record
VDV, fourth-power dose, report if VDV/(*a*_w_*T*^1/4^) > 1.75	VDV = (Σ*w*^4^Δ*t*)^1/4^, segments combined by the fourth-power rule
*A*(8) normalised to *T*_0_ = 28,800 s	*A*(8)^2^ accumulated per segment as *k*^2^Σ*w*^2^/(*f*_s_·*T*_0_), with *T*_0_ a firmware constant
Duration-dependent HGCZ reporting	On-node classification against configured guidance boundaries for deployment reporting, with duration-scaled *a*_w_ thresholds

^a^ *W*_k_: *f*_1_ = 0.4 Hz, *f*_2_ = 100 Hz, *f*_3_ = *f*_4_ = 12.5 Hz, *Q*_4_ = 0.63, *f*_5_ = 2.37 Hz, *f*_6_ = 3.35 Hz, and *Q*_5_ = *Q*_6_ = 0.91. *W*_d_: *f*_3_ = *f*_4_ = 2.0 Hz and *Q*_4_ = 0.63. ^b^ *w* denotes a frequency-weighted acceleration sample and *N* the sample count.

**Table 2:** Wired core evaluation: per-trial differences between the node and the reprocessed DT-178A reference (Z-axis), with the excitation in the one-third-octave band centred at 6.3 Hz. Difference = 100 × (CEM − node)/node.

Test	*k_z_*·*a*_w,*z*_ Diff (%)	*A_z_*(8) Diff (%)	VDV*_z_* Diff (%)	CF (Node)	CF (CEM)
08	−6.31	−9.23	9.24	6.25	9.06
09	−3.80	−3.78	6.82	6.71	7.06
10	−5.44	−5.43	1.90	6.91	6.68
11	−3.06	−3.05	15.76	6.14	10.91
12	3.31	3.32	17.22	7.82	8.65
13	10.18	10.19	31.25	8.72	10.68
14	−8.08	−8.07	−1.84	8.39	6.38
15	−11.42	−11.41	5.58	7.03	8.46
16	−0.46	−0.45	23.12	7.10	8.29
17	−15.91	−17.37	−6.09	6.83	6.78
Mean	−4.10	−4.53	10.30	—	—
SD	7.37	7.78	11.56	—	—

**Table 3:** Summary comparison of the wired sensing-and-computation core and the end-to-end wireless prototype against the reprocessed reference (Z-axis).

Quantity (Z-Axis)	Wired Core (Side Mount, *n* = 10)	Wireless Prototype (Stacked, *n* = 10)
Mean *A*(8) difference, % (95% CI)	−4.53 (−10.10 to 1.04)	−7.86 (−9.84 to −5.88)
Mean *k_z_*·*a*_w,*z*_ difference, % (95% CI)	−4.10 (−9.37 to 1.17)	−7.67 (−9.66 to −5.68)
SD of *A*(8) difference, %	7.78	2.77
Range of *A*(8) difference, %	−17.4 to +10.2	−10.86 to −1.62
Mean VDV difference, % (95% CI)	+10.30 (2.03 to 18.57)	−8.31 (−9.38 to −7.24)
SD of VDV difference, %	11.56	1.49
Pearson *r* (*k_z_*·*a*_w,*z*_) (95% CI)	0.945 (0.779–0.987)	0.994 (0.974–0.999)
Bland–Altman bias and 95% LoA (*k_z_*·*a*_w,*z*_), m/s^2^	−0.009 [−0.036, +0.018]	−0.109 [−0.195, −0.023]
Node crest factor (all trials)	<9	<9

Percentage differences are defined as 100 × (CEM − node)/node. Bland–Altman bias and limits of agreement are based on paired absolute differences in m/s^2^. Values in square brackets are the 95% limits of agreement. The wired and wireless sessions covered different excitation-amplitude ranges. The absolute Bland–Altman quantities are not directly comparable across configurations, and cross-configuration descriptions are based on the percentage quantities reported above.

**Table 4:** Payload-level data burden of raw acceleration streaming versus WBV exposure-metric telemetry.

Transmission Mode	Payload Basis	Approx. Sustained Rate
Raw 3-axis streaming	Nominal 1600 Hz × 3 axes × 2 B	≈9.6 kB/s
1 s diagnostic metrics	≈1 record/s, tens of bytes	≈10^1^–10^2^ B/s
60 s summary metrics	1 record/min	≈1 B/s
Segment-level exposure	Event-based (per segment)	≪1 B/s

## Data Availability

The data presented in this study are available on request from the corresponding author.
